# Biased Use of the *IGHV4* Family and Evidence for Antigen Selection in *Chlamydophila psittaci-*Negative Ocular Adnexal Extranodal Marginal Zone Lymphomas

**DOI:** 10.1371/journal.pone.0029114

**Published:** 2011-12-27

**Authors:** Daxing Zhu, Chen Lossos, Jennifer R. Chapman-Fredricks, Julie M. Matthews, Offiong F. Ikpatt, Phillip Ruiz, Izidore S. Lossos

**Affiliations:** 1 Division of Hematology-Oncology, Department of Medicine, University of Miami, Sylvester Comprehensive Cancer Center, Miami, Florida, United States of America; 2 Department of Pathology, University of Miami, Miami, Florida, United States of America; 3 Department of Molecular and Cellular Pharmacology, Sylvester Comprehensive Cancer Center, University of Miami, Miami, Florida, United States of America; University of Navarra, Center for Applied Medical Research, Spain

## Abstract

Extranodal marginal zone lymphomas (EMZL) are the most common lymphomas in the ocular adnexa. The etiology and potential role for antigenic stimulation in these lymphomas are still controversial. We have examined *IGHV* gene usage and mutations in 67 *Chlamydophila psittaci*-negative ocular adnexal EMZL. Clonal *IGHV* gene sequences were identified in 43 tumors originating from the orbit (19), conjunctivae (18) and lacrimal gland (6). Forty four potentially functional clonal *IGHV* gene sequences were detected with overrepresentation of the *IGHV4* family and *IGHV4-34* gene. All but 3 sequences were mutated with the average percent homology to the germ line of 93.5±6.1. Multinomial model and Focused binomial test demonstrated evidence for positive and/or negative antigen selection in 59% of the potentially functional *IGHV* genes. Intraclonal variation was detected in 8 of 11 tumor specimens. Overall our findings demonstrate that *C. psittaci*-negative ocular adnexal EMZL exhibit biased usage of *IGHV* families and genes with evidence for intraclonal heterogeneity and antigen selection in multiple tumors, implicating B-cell receptor-mediated antigen stimulation in the pathogenesis of these lymphomas.

## Introduction

Extranodal marginal zone lymphomas (EMZLs) are a distinct subtype of Non-Hodgkin's lymphoma (NHL) typically arising in extranodal sites devoid of significant lymphoid tissue, such as the gastrointestinal tract, thyroid and salivary glands, lungs, skin and the ocular adnexa (lacrimal gland, orbit, conjunctiva and eyelid) [1], [2]. EMZLs are frequently characterized by an indolent clinical course and often remain localized at their sites of origin for many years. In some locations, these lymphomas are preceded by the acquisition of reactive localized inflammatory infiltrates caused by long-standing chronic infection (e. g. *Helicobacter pylori* in the stomach) or autoimmune diseases (e.g. Hashimoto's thyroiditis in the thyroid and Sjogren's syndrome in the salivary glands) suggesting pathogenetic dependence on antigen stimulation and/or help provided by the local immune reactions [2], [3], [4], [5], [6]. Remission of early gastric EMZLs following *Helicobacter pylori-*eradicating antibiotics supports this mechanism of lymphoma pathogenesis [3], [4].

In contrast, the etiology and pathogenesis of ocular adnexal extranodal marginal zone lymphomas (OAEMZLs) are still controversial [7]. In a prospective case-controlled study from Italy, a significant association was demonstrated between exposure to household animals, rural residence, and history of chronic conjunctivitis in patients with OAEMZLs [8]. Furthermore, the Italian group and investigators from several other geographic regions demonstrated *Chlamydophila psittaci (C. psittaci*) DNA and bacteria present in OAEMZLs [7], [8], [9], [10], [11], [12], suggesting that this pathogen may be implicated in the development of these lymphomas analogously to *Helicobacter pylori* in gastric EMZLs. However, similar studies performed by us and other investigators on US and non-US-based patients have failed to corroborate the finding of *C. psittaci* in OAEMZLs, potentially suggesting geographic differences [7], [13], [14], [15], [16]. Furthermore, DNA from other bacteria was also not detected, which supports a non-bacterial etiology in Florida US patients [17].

The majority of B-cell lymphomas express a unique clonal surface B-cell receptor containing an immunoglobulin (Ig) heavy chain variable region (*IGHV*) that may have important implications for tumor diagnosis and pathogenesis. The following approaches can be useful to trace the developmental stage at which neoplastic B cell transformation occurs and provide support for B-cell receptor-mediated direct antigen stimulation of lymphoma cells without prior antigen identification: (1) examining possible bias in *IGHV*-gene usage; (2) analyzing the distribution of silent (S) and replacement (R) mutations in framework regions (FR) and complementary determining regions (CDR); (3) evaluating the CDR3 sequences; and (4) investigating the presence of intraclonal heterogeneity as a marker of ongoing somatic mutations. Several groups have analyzed the *IGHV* gene in small cohorts of patients (range 8–26) with OAEMZLs [18], [19], [20], [21], [22]; however, these studies have not yielded consistent results. To further elucidate the potential bias in *IGHV* gene usage and more robustly examine evidence for antigen selection, we analyzed *IGHV* gene sequences derived from fresh tumor samples in a large cohort of *C. psittaci*-negative, untreated OAEMZLs.

## Results

### Usage of *IGHV* genes in OAEMZLs

DNA extracted from fresh OAEMZL tumors from 67 patients (median age 63, range 24–92; 39 females and 28 males) was used for PCR amplification of the *IGHV* gene. The tumors originated in the orbit (29), conjunctiva (26), lacrimal gland (11) and eyelid (1). All specimens were negative for *C. psittaci*. Tumors from 34 of these 67 patients were used in our previous studies evaluating the presence of *C. psittaci* and other bacteria in OAEMZL [13], [17]. Agarose gel examination detected a PCR amplicon of the appropriate size for amplified *IGHV* in all 67 tumors. However, in 24 (36%) tumors, a clonal *IGHV-IGHJ* PCR sequence could not be identified, despite successful actin amplification, which served as a control for DNA integrity.

A total of 45 clonal *IGHV* sequences were detected in 43 patients, 23 females and 20 males, with a median age of 63 (range 39–92), representing 64% of the analyzed cohort. In two tumors two clonal sequences were detected in each tumor. Tumors with detected clonal sequences originated from the orbit (19), conjunctiva (18) and lacrimal gland (6). No differences in clinical features and tumor localization between patients with or without clonal *IGHV* sequences were detected. In 14 (32.6%) cases the PCR product could be sequenced directly, whereas in 29 (67.4%) cases, PCR amplicons had to be subcloned to identify the *IGHV-IGHJ* gene sequence.

A total of 44 identified clonal *IGHV* sequences were potentially functional (Table 1), while one sequence encoded by IGHV2-26*1 harbored an out-of-frame junction with a stop codon. This nonfunctional sequence was identified in a tumor that did not harbor another potentially functional clonal *IGHV* sequence. The 44 potentially functional *IGHV* were derived from 4 of the 7 human *IGHV* gene families with the following distribution: *IGHV*1, 11.4%; *IGHV*2, 2.2%; *IGHV*3, 40.9% and *IGHV*4, 45.5%. In comparison to the relative complexity of functional germline *IGHV* genes within each family and to the use of *IGHV* families in peripheral and lymph node lymphocytes in healthy donors [23], [24], [25], [26] (Table 2), the *IGHV* gene family usage by the OAEMZLs was biased with overrepresentation of the *IGHV*4 gene family (p = 0.001). The *IGHJ* segments in the potentially functional amplicons were derived from 5 of the 6 families with the following distribution: *IGHJ*2, 11.4%; *IGHJ*3, 4.5%; *IGHJ*4, 43.2%; *IGHJ*5, 22.7% and *IGHJ*6, 18.2%. Their usage was also significantly different from the reported repertoire in peripheral blood lymphocytes (p<0.001), with an over-representation of *IGHJ2* and *IGHJ5* [25].

**Table 1:** Molecular Analysis of Potentially Functional IGHV Genes in Patients with Ocular Adnexal Extranodal Marginal Zone Lymphomas.

Patient Number	Location	V Gene	J gene	V Gene Homology	FR R	FR S	CDR R	CDR S	P-Value FR Lossos	P-Value CDR Lossos	P-Value FR CLIP	P-Value CDR CLIP
2491	Conjuctiva	IGHV3-74*01	IGHJ4*02	97.5	3	0	3	1	0.16861	0.03427	0.389	0.0802
3050	Conjuctiva	IGHV1-3*01	IGHJ5*02	99.3	2	0	0	0	0.79635	0.61902	0.189	NA
3145	Orbit	IGHV3-11*01	IGHJ4*02	95.4	6	4	3	0	0	0.17078	−0.251	−0.5
3198	Orbit	IGHV4-34*02	IGHJ4*01	77.5	29	13	21	1	0.00233	0.00001	−0.00514	0.182
3687	Conjuctiva	IGHV3-11*01	IGHJ4*02	94.7	8	3	4	0	0	0.08868	−0.251	0.342
4277	Orbit	IGHV4-34*01	IGHJ4*02	93.6	8	3	5	2	0.06021	0.04022	−0.292	0.17
4438	Lacrimal	IGHV4-34*01	IGHJ6*02	100	0	0	0	0	NA	NA	NA	NA
4672	Orbit	IGHV4-59*01	IGHJ6*03	99.3	0	0	2	0	0.07201	0.00773	NA	0.0389
4694	Lacrimal	IGHV3-23*01	IGHJ5*02	90.9	10	8	5	3	0.00838	0.17802	−0.00616	−0.0477
4726	Orbit	IGHV4-34*01	IGHJ6*02	89.8	9	11	7	2	0.00031	0.04055	−1.50E-5	−0.303
4784	Lacrimal	IGHV4-4*07	IGHJ5*02	100	0	0	0	0	NA	NA	NA	NA
4942	Orbit	IGHV2-5*10	IGHJ3*01	95.1	10	2	1	1	0.76707	0.73414	−0.457	−0.192
4968	Conjuctiva	IGHV3-30*03	IGHJ6*02	94.7	8	2	4	1	0.2487	0.0845	0.408	0.195
5334	Conjuctiva	IGHV4-59*01	IGHJ5*02	96.4	4	1	4	1	0.08753	0.01566	−0.259	0.293
5547	Conjuctiva	IGHV1-69*01	IGHJ4*02	94	8	6	2	1	0.09378	0.50383	−0.0264	−0.12
5859	Orbit	IGHV3-66*02	IGHJ4*02	96.8	4	2	3	0	0.14258	0.05382	−0.442	0.179
5897	Orbit	IGHV4-39*01	IGHJ5*02	86.9	20	9	8	1	0.15982	0.11506	−0.106	−0.316
5984	Lacrimal	IGHV4-34*01	IGHJ4*02	90.1	14	7	7	0	0.0852	0.03391	0.389	0.0845
6092	Orbit	IGHV3-23*01	IGHJ4*02	93.4	5	5	8	1	0.00102	0.00095	−0.0116	0.386
6574	Conjuctiva	IGHV3-30*03	IGHJ4*03	97.2	8	0	0	0	0.98906	0.84086	0.367	−0.118
6901	Conjuctiva	IGHV1-24*01	IGHJ4*02	93.7	8	3	4	3	0.05443	0.12566	−0.0491	0.354
7126	Conjuctiva	IGHV4-b*02	IGHJ5*02	95.1	6	5	2	1	0	0.42527	−0.0979	−0.193
7513	Conjuctiva	IGHV3-66*01	IGHJ4*02	91.9	12	4	6	1	0.14813	0.03733	−0.21	0.459
7806	Lacrimal	IGHV1-46*01	IGHJ4*02	88.1	20	9	2	3	0.28245	0.88855	−0.0342	−0.012
11274	Orbit	IGHV3-23*01	IGHJ4*02	84	18	12	15	1	0.00093	0.00031	−0.00472	0.459
11498	Lacrimal	IGHV3-66*02	IGHJ6*02	99.3	1	1	0	0	0.37303	0.61621	−0.276	−0.208
11652	Orbit	IGHV4-4*02	IGHJ4*02	93	8	8	3	1	0.02871	0.39729	−0.0316	−0.123
12187	Orbit	IGHV3-23*01	IGHJ2*01	95.8	4	4	3	1	0.02615	0.12876	−0.0663	−0.463
12652	Conjuctiva	IGHV3-23*01	IGHJ4*02	95.1	4	5	4	1	0.00699	0.06246	−0.0405	0.4695
12823	Conjuctiva	IGHV4-34*02	IGHJ5*01	71.5	36	22	20	3	0.00041	0.00123	−5.33E 6	−0.2081
13051	Orbit	IGHV4-59*08	IGHJ5*02	96.1	5	4	1	1	0.1721	0.68027	−0.1522	−0.0972
13299	Orbit	IGHV3-30*16	IGHJ2*01	85.7	20	8	12	1	0.04391	0.00388	−0.1231	−0.4905
13299	Orbit	IGHV4-30-4*01	IGHJ4*03	99.3	2	0	0	0	0.815	0.6283	0.2318	NA
13726	Orbit	IGHV3-30-3*01	IGHJ6*02	96.1	1	5	5	0	0.00024	0.00554	−0.0063	0.2859
13928	Orbit	IGHV1-46*01	IGHJ5*02	91.6	12	5	5	2	0.08998	0.13444	−0.2314	0.4528
14394	Orbit	IGHV3-30*04	IGHJ6*02	98.2	2	1	1	1	0.17784	0.323	−0.2212	−0.4097
14425	Conjuctiva	IGHV4-30-4*01	IGHJ6*02	99.6	0	1	0	0	0.19586	0.56889	−0.1595	−0.3041
15137	Conjuctiva	IGHV4-34*03	IGHJ2*01	91.5	13	9	0	2	0.2006	0.97983	−0.078	−0.0039
15523	Conjuctiva	IGHV3-30*03	IGHJ4*02	99.6	0	1	0	0	0.18992	0.56667	−0.1526	−0.2941
15721	Orbit	IGHV4-34*01	IGHJ2*01	93.6	9	6	2	1	0.13703	0.53063	−0.0645	−0.1648
15721	Orbit	IGHV4-31*03	IGHJ2*01	99.6	0	0	1	0	0.19465	0.06769	NA	0.1943
16219	Orbit	IGHV4-30-4*03	IGHJ5*02	81	28	11	14	2	0.07453	0.01168	−0.0927	0.4631
16299	Orbit	IGHV4-39*01	IGHJ4*02	100	0	0	0	0	NA	NA	NA	NA
18664	Conjuctiva	IGHV3-74*01	IGHJ3*01	90.9	12	8	6	0	0.05611	0.09813	−0.0051	−0.2759

NA-not applicable.

**Table 2:** IGHV Family Usage in Ocular Adnexal Extranodal Marginal Zone Lymphomas.

IGHV Family	OAEMZLsPresent Study (n = 44)(%)	Relative IGHV Family Size(%) [23]	Adult PBL(in situ hybridization) (%) [24]	Adult PBL (single cell PCR) (%) [25]	Adult PBL (PCR)(%) [26]
IGHV1	11.4	21.5	11	12.7	12
IGHV2	2.2	5.9	3	4.2	1
IGHV3	40.9	43.1	52	56.3	55
IGHV4	45.5	21.6	19	19.7	26
IGHV5	0	3.9	10	5.6	4
IGHV6	0	2.0	5	1.4	2
IGHV7	0	2.0	0	0	0

OAEMZL- Ocular Adnexal Extranodal Marginal Zone Lymphomas.

PBL-peripheral blood lymphocytes.

The most frequently encountered genes were *IGHV*4-34 (n = 8), *IGHV*3-30 (n = 6) and *IGHV*3-23 (n = 5) (Table 1) which are known to frequently encode autoantibodies [25], [27], [28]. *IGHV*4-34 and *IGHV*3-30 were used at higher frequency than expected in normal individuals [25]. Specifically, the most commonly used gene, *IGHV*4-34, represented 18.2% of all the potentially functional *IGHV* genes identified in this study in contrast to its 3–9% prevalence in adult peripheral B lymphocytes [25], [29]. Similarly, *IGHV*4-34 was also detected once (3.2%) among 31 *IGHV* genes sequenced from normal adult marginal zone lymphocytes [30].

### Mutation Pattern and CDR3 Analyses of Potentially Functional *IGHV* genes

A total of 41 potentially functional clonal *IGHV* gene sequences harbored mutations, with 32 exhibiting more than a 2% difference from the most similar germline gene sequences and 9 sequences harboring a 2% or less difference. A total of 3 clonal *IGHV* gene sequences exhibited a germline sequence without mutations. The average percent homology to the germ line sequence in the 41 potentially functional mutated sequences was 93.5% (range 71.5–99.6). Eight of the 44 (18.2%) potentially functional *IGHV* gene isolates differed by more than 10% from the most similar germline counterpart. In the two tumors which each had two potentially functional *IGHV* gene isolates there was a 5 and 14% difference in the mutation load between the two sequences from the same tumor, respectively. Two of the three *IGHV*3-30 sequences harbored a 2% or less difference from the most similar germline genes, while all the *IGHV*3-23 and most *IGHV*4-34 sequences had more than a 2% difference from the most similar germline genes. OAEMZLs did not exhibit excessive accumulation of acceptor sequence motifs for *N*-glycan addition.

We next examined if the observed mutations in the OAEMZL *IGHV* regions tend to occur in the RGYW/WRCY sequences usually targeted by AID [31] and WA/TW sequences usually arising as a polymerase error during repair of the AID-generated lesions [32]. Overall, 51.7% of mutations observed in the OAEMZL cases occurred in AID target/error prone repair regions (30% and 21.7%, respectively), while only 23.4% and 17.7% of the germline genes consisted of areas subjected to AID action and error-prone repair, respectively. Overall, 7.9% of polymerase error-prone repair targets and 8.3% of AID targets were mutated as compared to 5.3% for the rest of the germline sequences, indicating a bias in mutation patterns toward mutational hotspots, as previously reported [31], [32].

To analyze for potential antigen selection pressure on the *IGHV* genes, we applied two algorithms: the Multinomial Model and Focused binomial test (Table 1). Both algorithms were used since presently there is no biologically confirmed method that precisely estimates antigen selection and each of these two most commonly used methods is associated with potential limitations: interaction between positive and negative selections in the multinomial model and underestimation of positive selection in the CDR regions by the Focused binomial test [33]. The Multinomial algorithm for antigen selection revealed evidence for positive selection manifested by an excess of replacement (R) mutations in the CDR exceeding that expected to occur by chance in 15 sequences. Negative selection manifested by a scarcity of R mutations in the FR was detected in 17 sequences. In 7 OAEMZL tumors a concomitant scarcity of R mutations in FRs and excess of R mutations in CDRs were observed. Selection analysis using the Focused binomial test demonstrated evidence of selection in the FR in 13 sequences and in the CDR in 4 sequences. The markedly smaller number of cases with selection in the CDR by the Focused binomial test as compared to the Multinomial model could be predicted, since the former test is known to underestimate positive selection in the CDR [33]. Selection was detected in all 5 *IGHV*3-23 and 6 of the 8 functional *IGHV*4-34 sequences by either of the two models.

We performed a search for similar R mutations within the CDRs of isolates belonging to the same *IGHV* genes (Table 3). Recurrent amino acid changes were observed in the CDR1 and CDR2 of the *IGHV4-34*, *IGHV3-30* and *IGHV3-23* sequences. Repeated mutations in the CDR2 region resulting in a loss of serine from the germline region were observed in all three genes, and were replaced with aspartic acid, glycine, alanine, threonine, and asparagine, respectively. Additional observed mutations included changes of isoleucine to valine, asparagine to serine, and threonine to alanine. The prevalence of recurrent amino acid changes was higher in the CDR2 region than the CDR1 region, suggesting that the CDR2 region may markedly contribute to the paratope of the tumor-derived Ig. Next we compared mutation patterns present in the CDR2 of the *IGHV4-34* from our 8 OAEMZL tumors to 70 sequences originating from normal peripheral blood, tonsils and marginal zone B cells identified in GenBank (accession numbers are shown in the Table S1). Except for recurrent mutations of serine in the second residue that were observed in both normal B cell and OAEMZL sequences, recurrent mutations of isoleucine in the first residue to valine, serine in the forth and sixth residues to aspartic acid and glycine, respectively, and threonine in the seventh residue to alanine were not observed in normal B cells (data not shown), suggesting that the mutations observed in the OALMZL may be tumor antigen specific. However, future analysis of additional sequences will be required to confirm this observation.

**Table 3:** Repeating Mutations in CDR1 and CDR2.

Germline	Case Number	Site	CDR1	CDR2
**IGHV4-34**				
	15137	Conjuctiva	GGSFSGYY	INHSGST
	4438	Lacrimal	GGSFSGYY	INHSGST
	15721	Orbit	GGSFSAYY	INHSG**G**T
	4726	Orbit	GGSFTGYY	INYAGDP
	4277	Orbit	GGS**L**SGYY	I**S**H**D**GS**A**
	5984	Lacrimal	GGSFSGYF	**V**DPSGR**A**
	12823	Conjuctiva	GGP**L**G**T**NI	**VS**ENGET
	3198	Orbit	GDYFI**T**YA	**VS**Q**D**A**G**V
	Germline		GGSFSGYY	INHSGST
**IGHV3-30**				
	15523	Conjuctiva	GFTFSSYG	ISYDGSNK
	6574	Conjuctiva	GFTFSSYG	ISYDGSNK
	14394	Orbit	GFTFSSYA	ISYDGSNK
	13726	Orbit	RFTFS**N**YA	ISYNGNNQ
	4968	Conjuctiva	GFIFS**N**YG	ISYDG**T**IK
	13299V3	Orbit	GFTLSSHG	SSFDG**T**TE
	Germline		GFTFSSYG	ISYDGSNK
**IGHV3-23**				
	4694	Lacrimal	GFTFSNYA	ISGSGS**N**T
	6092	Orbit	GFTFSRYV	IS**A**SGS**N**T
	12187	Orbit	GFTFT**T**YA	I**G**GSGGST
	12652	Conjuctiva	GFTFS**T**YA	I**G**GSGGVT
	11274	Orbit	TITFNCCA	IA**A**SGTGT
	Germline		GFTFSSYA	ISGSGGST

Repeated amino acid substitutions are shown in bold.

Analysis of the tumor-derived CDR3 sequences revealed low similarity and an absence of stereotyped sequences with no homology to antibacterial and other previously published antibodies. The average CDR3 isoelectric point was 5.95±1.92 (SD). The average CDR3 length was 15.75±3.67 (SD) amino acids, with 19 sequences harboring 10–14 amino acids, 18 sequences harboring 15–19 amino acids and 7 sequences of 20 or more amino acids.

In 22 patients, consisting of 6 cases with 2% or less difference from the most similar germline gene sequences and 16 cases with more than 2% difference from the most similar germline gene sequences, information on clinical presentation and outcome following localized radiation to the eyes was available with a median follow up of 31 months (range 2–121). There was no difference in overall survival and progression free survival between these two groups of patients (not shown); however, the small number of cases and clinical events prevents firm conclusions.

### Analysis of intraclonal heterogeneity

**Figure 1:**
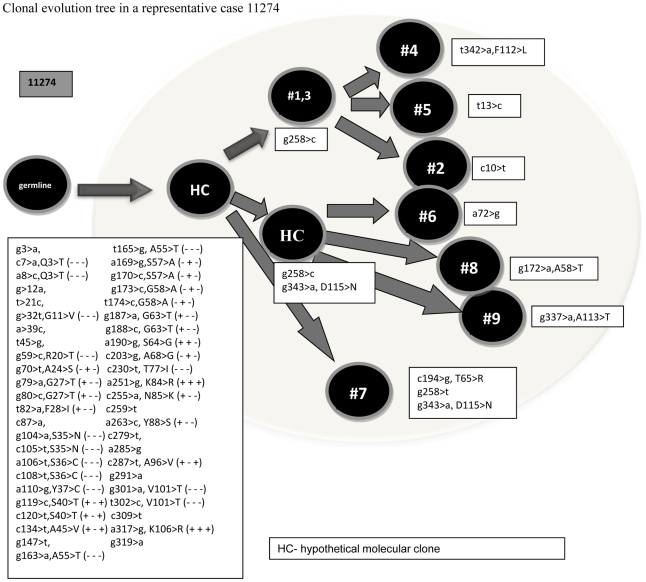
Clonal evolution tree in a representative case 11274. HC- hypothetical molecular clone.

To assess the presence of intraclonal heterogeneity, extensive molecular cloning of 11 randomly selected potentially functional *IGHV* gene isolates with percent homology to the germ line sequence ranging from 81.0 to 94.7 was performed (Table 4 and Figure 1). In three of the tested samples, the extensively mutated clonal *IGHV* gene isolates did not show intraclonal heterogeneity. In 8 samples intraclonal heterogeneity was detected as manifested by the presence of molecular clones harboring confirmed mutations not observed in the most abundant clonal *IGHV* gene sequence. While single nucleotide mutations accounted for most of the observed ongoing mutations, deletions were also observed. The extent of intraclonal heterogeneity was limited in 5 of these cases with 1 or 2 additional confirmed mutations, similar to our previous observation in a subset of diffuse large B-cell lymphoma cases [34]. In two tumors (4694 and 13299), 16 and 56 additional confirmed mutations were observed, thus leading to extensive variation between the subclones harboring identical CDR3 regions, similar to intraclonal heterogeneity which is typically observed in follicular lymphoma [35].

**Table 4:** Analysis of Intraclonal Heterogeneity in Ocular Adnexal Extranodal Marginal Zone Lymphomas.

Case Number	Site of origin	Most Similar Germline VH Gene	Total No. of Molecular Clones Evaluated	No. of tumor-derived Molecular Clones	No. of Confirmed Additional Mutations	No. of Single Unconfirmed Mutations
3687	Conjunctiva	IGHV3-11*01	11	9	4	4
4694	Lacrimal Gland	IGHV3-23*01	14	14	16	8
4726	Orbital	IGHV4-34*01	14	14	0	7
5547	Conjunctiva	IGHV1-69*01	21	20	1	6
6092	Orbital	IGHV3-23*01	13	13	0	6
11274	Orbital	IGHV3-23*01	9	9	1	8
11652	Orbital	IGHV4-4*02	12	12	2	3
13051	Orbital	IGHV4-59*08	11	11	2	2
13299	Orbital	IGHV4-30*01	26	22	56*	9
16219	Orbital	IGHV4-30*03	11	7	1	8
18664	Conjunctiva	IGHV3-74*01	24	24	0	26

*The 56 mutations include 52 single nucleotide changes and 4 deletions ranging from 1–9 base pairs all resulting in potential functional protein.

## Discussion

OAEMZLs account for up to 55% of all orbital tumors and 8% of extranodal NHLs [7]. While antigen stimulation, which may be directly mediated by surface B-cell receptor and/or indirectly mediated by T cells, is implicated in the pathogenesis of EMZLs in the thyroid, salivary gland and stomach, the etiology of OAEMZL is still controversial. *C. psittaci* may function as the antigenic stimulus of OAEMZLs in several geographic regions [7], [8], [11], [12]; however, it is not detected in the majority of US patient tumors, in which the antigen is unknown [13], [16], [36]. Overall, there is only scarce published data on the potential role of antigenic stimulation in this lymphoma.

The present study was undertaken to clarify if there is potential antigen stimulation in *C. psittaci*-negative OAEMZLs. To this end, molecular analysis of the *IGHV* region was performed in 67 untreated primary OAEMZL patients. The study demonstrates a biased usage of *IGHV* families and genes with evidence for intraclonal heterogeneity and antigen selection in multiple tumors, implicating B-cell receptor-mediated antigen stimulation in the pathogenesis of these lymphomas.

In our cohort of *C. psittaci*-negative OAEMZL patients there was overrepresentation of the *IGVH4* family and specifically the *IGVH4-34* gene segment. Similar biased usage of the *IGVH4-34* gene segment was previously suggested by Bahler et al. in a small cohort of *C. psittaci*-negative OAEMZL patients from the US [18]. In this study the *IGVH4-34* gene segment was detected in 3 of the 10 analyzed cases. In contrast, 4 small studies from Germany and Japan, analyzing a total of 56 tumors (range 8–26) did not demonstrate biased usage of the *IGVH4* family and *IGVH4-34* gene segment [19], [20], [21], [22]. In these 4 studies the *IGVH3* family (64%) was most commonly used. Overrepresentation of *IGHV*3-30, seen in our cohort, was not observed in these studies. *IGHV*3-23, commonly used in our cohort, was detected in 7 of these 56 cases but was overrepresented only in a study by Adam et al., accounting for 3 out of the 8 cases [22]. While all the analyzed US cases in both studies were negative for *C. psittaci*, its presence was not examined in any of the 4 non-US studies [19], [20], [21], [22]. In a recently accepted, but still not published manuscript that mostly analyzed patients from European countries, Dagklis et al. also did not observe biased usage of the *IGVH4* family and *IGVH4-34* gene segment [37]. Careful re-evaluation of the data in this manuscript discloses that *IGVH4-34* gene segment was not detected in any of the 15 *C. psittaci*-positive OAEMZL tumors, but was observed in 3 of the 21 of the tested *C. psittaci*-negative OAEMZL. The observed differences in the use of the *IGVH* families and genes between the US and non-US studies most probably stem from different antigens driving the lymphomagenesis of OAEMZL in distinct geographic locations, but the small number of analyzed cases, usage of archived material as a DNA source and differences in PCR methodologies might also contribute to the observed discrepancies. Further studies of *IGHV* in OAEMZL patients with and without *C. psittaci* from different geographic regions using DNA extracted from fresh or frozen tumors will help to clarify the observed discrepancy.

The gene most commonly used by the *C. psittaci*-negative OAEMZL in our patients as well as patients reported by Bahler et al [18] was *IGVH4-34*, accounting for 18.2% of the functional *IGVH* genes in our cohort. In healthy individuals, IGVH4-34 expressing cells are predominantly identified in naïve lymphocytes and are underrepresented in the germinal center and memory compartments [27], being used by 3–9% of adult peripheral B lymphocytes [25], [27], [29] and in one of 31 *IGHV* genes sequenced from normal adult marginal zone lymphocytes [30]. In lymphomas, the *IGVH4-34* gene is overrepresented in chronic lymphocytic leukemia cases with mutated *IGVH* genes (20%) [38] and in primary central nervous system diffuse large B-cell lymphomas (DLBCL) (60%) [39]. It is not overrepresented in patients with non- OAEMZLs. In its germline configuration, without contribution of somatic mutation and to a large extent independently of the CDR3 region and of associated light chains, *IGVH4-34* frequently encodes intrinsically autoreactive antibodies that recognize the I/i erythrocyte determinants constituting the antigenic target of pathogenic autoantibodies in cold agglutinin disease [40]. The *IGVH4-34* gene has also been reported to encode antibodies recognizing auto- allo- and exogenous antigens, such as DNA, Rh, cardiolipin and lipid-A and is overrepresented in patients with systemic lupus erythematosus [28], [41]. Two other genes (*IGVH3-30* and *IGVH3-23*) frequently used by OAEMZLs analyzed in the present study also frequently encode autoantibodies [25].

The erythrocyte I/i antigens bind to the FR1 region of the IGVH4-34 [40], a characteristic attributed to B-cell superantigens, which are supposed to directly activate B cells [42], [43], [44]. Among the *IGVH4-34* genes cloned from the OAEMZLs analyzed in the present study, one sequence was not mutated, 3 acquired similar mutations in the CDR2 and 3 preserved FR sequences, suggesting a possible role of a yet unknown B cell superantigen that might drive proliferation and lymphoma development.

The majority (93%) of the potentially functional *IGVH* genes isolated from the OAEMZLs analyzed in this study were mutated as compared to their germline counterparts, suggesting their origin from a cell that had experienced antigen selection during the germinal center reaction. This finding is concordant with previous reports, which also demonstrated somatic mutations in the majority of EMZLs irrespective of the anatomical site of origin [45], [46]. The observed level of mutations (mean percent homology to germline of 93.5±6.1) is also similar to the mutational load previously reported in both ocular adnexal and other EMZLs [18], [19], [20], [22], [45], [46]. However, 15% of the potentially functional *IGHV* gene isolates in the current study differed by more than 12% from the most similar germline counterparts- an uncommon observation in EMZLs irrespective of the anatomical site of origin. This extensive mutational load together with the evidence for positive and/or negative antigen selection (Table 1) in 59% of the potentially functional *IGHV* genes by either Multinomial Model and/or Focused binomial test further suggests that antigens play a role in the pathogenesis of these lymphomas. The prevalence of *IGHV* genes with negative selection may suggest that Ig preservation may be required for preventing the disruption of antigen-mediated signaling that could be harmful to lymphoma cell survival. Observation of intraclonal heterogeneity in the majority of the analyzed OAEMZLs using a very conservative definition in this study that required the presence of confirmed ongoing mutations, may further point to the existence of continuous antigen stimulation in their pathogenesis. Since EMZLs are in most cases considered to be of post germinal center origin, the presence of intraclonal heterogeneity in these tumors may reflect aberrant activation of the somatic mutational machinery or re-entry into germinal centers for further mutations. Indeed, colonization of germinal centers is commonly observed in EMZL biopsies [1] and may reflect re-entry of malignant cells into germinal centers for additional rounds of antigen selection.

Our methodological approach failed to identify clonal *IGHV-IGHJ* PCR product in 36% of tumors that were clinically and pathologically similar to the tumors in which clonality was determined. To prevent misidentification of the lymphoma *IGHV* genes, we used very strict criteria to define clonality which may account for some of the observed failures. However, previous studies reported PCR detection of a monoclonal population in only 25–70% of EMZL cases [47]. The inability to detect monoclonal *IGHV-IGHJ* gene sequences in these cases may result from the absence of *Ig* rearrangement, as is rarely observed in lymphomas [48]. We could not amplify the *IGHV-IGHJ* gene sequences of some of these tumors using the BIOMED-2 primers, while tumor clonality was confirmed by light chain amplification using the same protocol. Alternatively, somatic mutations in the region to which the PCR primers are designed to hybridize may lower amplification efficiency and lead to possible false-negative results.

Overall our findings demonstrate that *C. psittaci*-negative OAEMZLs exhibit biased usage of *IGHV* families and genes with evidence for intraclonal heterogeneity and antigen selection in multiple tumors, implicating B-cell receptor-mediated antigen stimulation in the pathogenesis of these lymphomas. The nature of the antigens that potentially play a role in these processes is currently unknown and requires further studies.

## Materials and Methods

### Patient material

DNA was extracted from a total of 67 patient fresh biopsy samples at the time of diagnosis (between 1991 and 2011) of OAEMZLs using a commercially available kit (QIAamp; Qiagen, Valencia, CA, USA), as described by the manufacturer. All pathologic specimens were classified according to the WHO 2008 classification on the basis of the morphologic features observed on routinely prepared hematoxylin and eosin–stained slides of formalin-fixed, paraffin-embedded tissues along with immunophenotypic and genotypic results [1]. Flow cytometry immunophenotyping was performed in most cases and clonality was confirmed by either Southern blot analysis or polymerase chain reaction (PCR) for immunoglobulin heavy or light chains in accordance with the BIOMED-2 recommendations [49]. This study was approved by the University of Miami Institutional Review Board (IRB) and written informed consent was obtained according to the approved protocol.

### Detection of *C. psittaci* DNA

Touchdown enzyme time-release polymerase chain reaction (PCR) for detection of *C. psittaci* DNA was performed as previously reported by us [13]. Blank reactions were always run concomitantly with the DNA from patients' specimens to monitor for possible contamination of PCR reagents and to rule out false-positive results. All reactions were repeated twice.

### PCR amplification, cloning and sequencing of *IGHV* genes

To amplify the *IGHV-IGHJ* gene sequences, 50–200 ng of DNA were amplified by GoTaq Green Master Mix (Promega, Madison, WI) in a final volume of 50 µl containing 10 pmol of a specific 5′ primer corresponding to one of the 6 human variable immunoglobulin heavy chain family leaders (*IGHV*1 through *IGHV*6) and 10 pmol of 3′ antisense J_H_ consensus primer [48], [50]. *IGHV*1 leader primer also amplifies sequences from the closely related *IGHV*7 family. The PCR conditions were: 96°C for 5 minutes, 55°C for 1 minute, 72°C for 3 minutes, 1 cycle; 94°C for 30 seconds, 55°C for 30 seconds, 72°C for 30 seconds, 30 to 35 cycles; and 72°C for 7 minutes. A control with no added template was used in each PCR reaction to exclude the possibility of contamination. DNA integrity from patient samples was verified by amplification of ß-actin using specific PCR primers yielding a 597 base pair (bp) amplicon, as described previously [17]. PCR products were analyzed by 2% agarose gel electrophoresis and stained with ethidium bromide. All bands of the appropriate size were excised from the gels and purified by adsorption to a silica matrix (QIAquick Gel Extraction Kit, Qiagen). Direct DNA sequencing of PCR amplicons was performed on a 373 automatic DNA sequencer (Applied Biosystems, Foster City, CA) using the ABI Prism Big Dye Terminator Kit (Perkin Elmer, Foster City, CA), as recommended by the manufacturer. The same primers used for the PCR were used for sequencing. If a direct DNA sequencing attempt of the PCR amplicon failed to recover an unambiguous sequence, the PCR amplicons were cloned using a TOPO TA cloning kit and OneShot TOP10' chemically competent E. coli cells (Invitrogen, Carlsbad, CA) according to the manufacturer instructions. The colony direct PCR assay was used to determine whether colonies included the correct PCR insert. At least 4 colonies from two independent PCRs were sequenced. Sequences were analyzed with the ImMunoGeneTics V-Quest (IMGT V-QUEST) software (http://www.imgt.org). The sequences were defined as clonal if identical CDR3 sequences were obtained from 2 independent PCR reactions by direct sequencing. If cloning was performed, at least two sequences with identical CDR3 from each of the two independent PCRs (a total of 4 identical sequences) were required for definition of clonal sequence. All the clonal sequences were deposited in GenBank (JN646046-JN646089).

### Analysis of the *IGHV* sequences

Sequences were compared with known germline genes and assigned *IGHV* and *IGHJ* germline sequence based on the highest percentage of sequence homology [48]. The length of the CDR3 region and number of somatic mutations were determined. Mutations at the last nucleotide position of the sequenced fragment were excluded from the mutational analysis because they might result from nucleotide deletion at the joining sites [48]. The percent of sequence identity was calculated from the aligned sequences from the beginning of FR1 to the end of FR3. The total number of mutations per variable region (FR1-FR3) were added and normalized to the respective length of each region according to the IMGT numbering scheme to analyze the relative frequency of mutations in the different variable regions. The number of coding (replacement-R) and non-coding (silent-S) mutations were obtained from IMGT V-QUEST tabulated data. Antigen selection analysis was performed using the Multinomial Model [51] and the Z-Test version of the Focused binomial test [52]. For analysis of recurrent amino acid mutations in the CDR1 and CDR2 regions, sequences derived from the same germline *IGVH* gene in 4 or more specimens were aligned to their corresponding IMGT assigned germline CDR1 and CDR2 regions by ClustalW (http://www.ebi.ac.uk/Tools/msa/clustalw2/). To determine the CDR3 isoelectric point, the pI/MW application of the Swiss-Prot/TrEMBL software was used (http://web.expasy.org/compute_pi/).

Somatic hypermutations tend to occur at hotspots, such as RGYW/WRCY sequences usually targeted by the mutagenic enzyme AID [31] and WA/TW sequences usually associated with mutations in the A:T pairs considered to arise as copying errors introduced by a DNA polymerase during repair of AID-generated lesions [32]. To analyze for potential mutation enrichment in these sequences, we have 1) calculated the percentage of mutations at these hotspots relative to the total number of mutations observed in all the analyzed sequences, and 2) examined the prevalence of mutations in these motifs by normalizing to the relative length of these hypermutational hotspots across all the clonal germline sequences detected in the analyzed cohort.

To determine whether homology existed between patients' CDR3 to the CDR3 of previously reported Ig genes, the amino acid sequence of patients' CDR3 region was run through IgBLAST (http://www.ncbi.nlm.nih.gov/igblast/) and TEIRESIAS algorithm (http://cbcsrv.watson.ibm.com/download.phtml.html). The previously reported parameters, enabling the connection of all pairs of sequences that shared at least 50% amino-acid identity and 70% similarity, were used for this analysis [53].

### Analysis of intraclonal heterogeneity

Intraclonal heterogeneity was examined in 11 ocular adnexa MALT lymphoma specimens by repeated cloning and sequencing of at least 9 tumor-derived molecular clones from each specimen. For evaluation of intraclonal heterogeneity, we used previously proposed definitions [34], [48]: confirmed mutation- a mutation observed more than once in the *IGHV* gene molecular clones from the same tumor specimen; unconfirmed mutation- a substitution mutation observed in only 1 of the *IGHV* gene molecular clones from the same tumor specimen. Only the confirmed mutations were considered as evidence of intraclonal heterogeneity while the unconfirmed mutations, which may result from Taq polymerase errors were disregarded.

### Statistical Analysis

The usage of the *IGHV and IGHJ* gene segments in the ocular adnexa MALT lymphoma specimens was compared to their utilization in normal peripheral blood lymphocytes by the X^2^ test, with p<0.05 defined as statistically significant. The Multinomial Model and the Focused binomial test were applied as previously reported [51], [52].

## Supporting Information

Table S1 **Accession information of 70 VH4-34 sequences originating from normal peripheral blood, tonsil, and marginal zone B cells that were used for comparison with the OAEMZL tumor sequences.**
(DOC)
