# Supplementary material for: Biased Use of the IGHV4 Family and Evidence for Antigen Selection in Chlamydophila psittaci-Negative Ocular Adnexal Extranodal Marginal Zone Lymphomas
Source: PLoS One. 2011 Dec 27;6(12):e29114. doi: 10.1371/journal.pone.0029114 (PMC3246466; doi:10.1371/journal.pone.0029114)
Supplement: Table S1 — Accession information of 70 VH4-34 sequences originating from normal peripheral blood, tonsil, and marginal zone B cells that were used for comparison with the OAEMZL tumor sequences. (DOC) [file pone.0029114.s001.doc]

Table S1

Accession information of 70 VH4-34 sequences originating from normal peripheral blood, tonsil, and marginal zone B cells that were used for comparison with the OAEMZL tumor sequences

SEQUENCE ID

| gi|773607|gb|U21257.1|HSU21257_Human_rearranged_I |
| --- |
| gi|773627|gb|U21267.1|HSU21267_Human_rearranged_I |
| gi|773629|gb|U21268.1|HSU21268_Human_rearranged_I |
| gi|794038|emb|X84166.1|_H.sapiens_Ig_heavy_chain |
| gi|794039|emb|X84167.1|_H.sapiens_Ig_heavy_chain |
| gi|794040|emb|X84168.1|_H.sapiens_Ig_heavy_chain |
| gi|794052|emb|X84180.1|_H.sapiens_Ig_heavy_chain |
| gi|1052685|emb|X87076.1|_H.sapiens_B8-g4C02_gene |
| gi|1052692|emb|X87085.1|_H.sapiens_B8-g4H10_gene |
| gi|1154751|emb|Z68463.1|_H.sapiens_rearranged_IgG |
| gi|1154754|emb|Z68466.1|_H.sapiens_rearranged_IgG |
| gi|1197299|emb|Z47229.1|_H.sapiens_mRNA_for_immun |
| gi|1197304|emb|Z47234.1|_H.sapiens_mRNA_for_immun |
| gi|1197305|emb|Z47228.1|_H.sapiens_mRNA_for_immun |
| gi|1197319|emb|Z47241.1|_H.sapiens_mRNA_for_immun |
| gi|1771018|emb|Z80707.1|_H.sapiens_BF2N1-g4A03_ge |
| gi|1771021|emb|Z80710.1|_H.sapiens_BF2N1-g4B10_ge |
| gi|1771033|emb|Z80722.1|_H.sapiens_BF2N1-g4F09_ge |
| gi|1771036|emb|Z80725.1|_H.sapiens_BF2N1-g4G03_ge |
| gi|1771045|emb|Z80737.1|_H.sapiens_BF2N2-g4D08_ge |
| gi|1771047|emb|Z80740.1|_H.sapiens_BF2N2-g4F05_ge |
| gi|1771059|emb|Z80755.1|_H.sapiens_BF2N3-g4F03_ge |
| gi|1791072|gb|U80115.1|HSU80115_Human_immunoglobu |
| gi|1869923|emb|Z80747.1|_H.sapiens_BF2N2-g4H04_ge |
| gi|2598474|emb|AJ222559.1|_Homo_sapiens_mRNA_for |
| gi|2598475|emb|AJ222560.1|_Homo_sapiens_mRNA_for |
| gi|2598476|emb|AJ222561.1|_Homo_sapiens_mRNA_for |
| gi|2598477|emb|AJ222562.1|_Homo_sapiens_mRNA_for |
| gi|2598478|emb|AJ222547.1|_Homo_sapiens_mRNA_for |
| gi|2598479|emb|AJ222548.1|_Homo_sapiens_mRNA_for |
| gi|2598480|emb|AJ222549.1|_Homo_sapiens_mRNA_for |
| gi|2598481|emb|AJ222550.1|_Homo_sapiens_mRNA_for |
| gi|2598482|emb|AJ222551.1|_Homo_sapiens_mRNA_for |
| gi|2598483|emb|AJ222552.1|_Homo_sapiens_mRNA_for |
| gi|2598484|emb|AJ222553.1|_Homo_sapiens_mRNA_for |
| gi|2598485|emb|AJ222554.1|_Homo_sapiens_mRNA_for |
| gi|2598486|emb|AJ222555.1|_Homo_sapiens_mRNA_for |
| gi|2598487|emb|AJ222556.1|_Homo_sapiens_mRNA_for |
| gi|2598488|emb|AJ222557.1|_Homo_sapiens_mRNA_for |
| gi|2598489|emb|AJ222558.1|_Homo_sapiens_mRNA_for |
| gi|2598490|emb|AJ222563.1|_Homo_sapiens_mRNA_for |
| gi|2598491|emb|AJ222564.1|_Homo_sapiens_mRNA_for |
| gi|2598492|emb|AJ222565.1|_Homo_sapiens_mRNA_for |
| gi|2598493|emb|AJ222566.1|_Homo_sapiens_mRNA_for |
| gi|2598494|emb|AJ222567.1|_Homo_sapiens_mRNA_for |
| gi|2598495|emb|AJ222568.1|_Homo_sapiens_mRNA_for |
| gi|2598496|emb|AJ222569.1|_Homo_sapiens_mRNA_for |
| gi|2598497|emb|AJ222570.1|_Homo_sapiens_mRNA_for |
| gi|2598498|emb|AJ222571.1|_Homo_sapiens_mRNA_for |
| gi|2598500|emb|AJ222573.1|_Homo_sapiens_mRNA_for |
| gi|2598501|emb|AJ222574.1|_Homo_sapiens_mRNA_for |
| gi|2598502|emb|AJ222575.1|_Homo_sapiens_mRNA_for |
| gi|2598503|emb|AJ222576.1|_Homo_sapiens_mRNA_for |
| gi|2598504|emb|AJ222577.1|_Homo_sapiens_mRNA_for |
| gi|2598505|emb|AJ222578.1|_Homo_sapiens_mRNA_for |
| gi|2808871|emb|AJ225703.1|_Homo_sapiens_DNA_for_I |
| gi|2808875|emb|AJ225705.1|_Homo_sapiens_DNA_for_I |
| gi|2808939|emb|AJ225742.1|_Homo_sapiens_DNA_for_I |
| gi|2808944|emb|AJ225745.1|_Homo_sapiens_DNA_for_I |
| gi|2808961|emb|AJ225755.1|_Homo_sapiens_DNA_for_I |
| gi|2809024|emb|AJ225685.1|_Homo_sapiens_DNA_for_I |
| gi|3646167|emb|AJ231572.1|_Homo_sapiens_rearrange |
| gi|3646204|emb|AJ231609.1|_Homo_sapiens_rearrange |
| gi|3646220|emb|AJ231625.1|_Homo_sapiens_rearrange |
| gi|4323661|gb|AF103029.1|_Homo_sapiens_isolate_do |
| gi|4323685|gb|AF103041.1|_Homo_sapiens_isolate_do |
| gi|4837780|gb|AF103122.1|_Homo_sapiens_clone_N124 |
| gi|4837882|gb|AF103173.1|_Homo_sapiens_clone_N186 |
| gi|4838092|gb|AF103278.1|_Homo_sapiens_clone_N78 |
| gi|8249652|emb|AJ289341.1|_Homo_sapiens_partial_I |
|  |
